# Inadvertent treatment of hypoadrenalism with prednisolone in pemphigus: A case report

**DOI:** 10.1002/ccr3.2132

**Published:** 2019-04-08

**Authors:** Sirazum Choudhury, Pratibha Machenahalli, Tricia Tan, Karim Meeran

**Affiliations:** ^1^ Section of Investigative Medicine, Division of Diabetes, Endocrinology and Metabolism Imperial College London London UK; ^2^ Department of Clinical Biochemistry Imperial College Healthcare NHS Trust London UK; ^3^ Endocrinology Imperial College Healthcare NHS Trust London UK

**Keywords:** adrenal insufficiency, glucocorticoid replacement, hypoadrenalism, prednisolone

## Abstract

Pituitary and adrenal insufficiency must not be overlooked when weaning patients down from high‐dose steroids. Prednisolone can be used as glucocorticoid replacement therapy, with most patients needing 3‐4 mg once daily.

## 1 INTRODUCTION

A 49‐year‐old male with hypopituitarism and pemphigus receiving 10‐30 mg prednisolone had unexpected improvement and was weaned to 4 mg but no lower due to tiredness and nausea. This experience highlights that the prednisolone dose required for adrenal replacement is 3‐4 mg daily, and hypothalamo‐pituitary‐adrenal failure should not be overlooked in patients on steroids.

The gradual reduction of prednisolone dosage in a patient with pituitary failure is an experiment that is never knowingly undertaken, because of the appropriate fear of an Addisonian crisis. As a consequence, there is an absence of good data on the minimum dose of prednisolone that can be used as a replacement.

This case highlights a patient who inadvertently became aware of his own glucocorticoid requirement. During this process, he has highlighted that once‐daily prednisolone is a convenient option for steroid replacement therapy, and that the doses required for adequate replacement are lower than previously thought. This case has changed local practice, and we now use prednisolone 2‐4 mg once daily and titrate the dose against a prednisolone level in patients with adrenal insufficiency.

## 2 CASE REPORT

A 49‐year‐old bus driver presented in November 2000 with features of acromegaly. An MRI scan demonstrated a large (2.0 × 2.0 × 0.5 cm) pituitary adenoma. His growth hormone levels were 14.8‐16.4 mU/L and were not suppressible with glucose. His IGF1 was 191 nmol/L (reference range: 13‐64 nmol/L), prolactin 6557 mU/L, testosterone 2 nmol/L, and cortisol uninterpretable given long‐term high‐dose prednisolone therapy for long‐standing pemphigus vulgaris. He was treated with a transsphenoidal hypophysectomy in January 2001 and external beam pituitary radiotherapy in October 2001. In the same year, he was discovered to be thyrotoxic, secondary to a toxic adenoma, which was treated with radioactive iodine. He has never required thyroid replacement therapy, and his current thyroid function tests 18 years later reveal that he is euthyroid (TSH 2.69 mU/L; free T4 10.6 pmol/L; free T3 3.5 pmol/L). He was noted to be osteopenic on a DEXA scan in 2004, prompting treatment with alendronate 70 mg weekly. Subsequent DEXA scans have demonstrated osteoporosis due to a combination of testosterone deficiency and long‐term high‐dose prednisolone treatment. Over the years, the patient was treated with cabergoline and later, octreotide in 2003, for excess levels of growth hormone and IGF‐1. These treatments were stopped in 2004, when his growth hormone level was found to be suppressed and the radiotherapy had clearly worked. Since 2001, he has required exogenous testosterone.

The patient had a pre‐existing diagnosis of pemphigus vulgaris which was treated with long‐term prednisolone, at doses no lower than 10 mg for over 15 years, and azathioprine 125 mg daily. His cortisol reserve was never assessed as it was thought unlikely that he would ever come off his high‐dose prednisolone. The patient reported that his skin “started burning” when the dose of prednisolone was ever reduced below 10 mg. He stayed on prednisolone for at least another 10 years and was regularly reviewed by the dermatologists.

Unexpectedly, by August 2014, the patient's pemphigus had undergone complete remission. He was very slowly weaned down on prednisolone according to a standard dermatological protocol (reduction by 1 mg every 2 months, with regular review for resurgence of skin inflammation), with a view to stopping completely. By October 2015, the patient had come off azathioprine completely and was taking prednisolone 3 mg daily, on which he was well. Unaware that the prednisolone served as glucocorticoid replacement, the patient continued to wean down to 2 mg, on which he experienced severe lethargy, and then 1 mg, on which he started vomiting, although he never had a salt‐losing Addisonian crisis. He independently up‐titrated the prednisolone to 4 mg once daily. A short synacthen test was performed in May 2016, the results of which were suggested that recovery of the cortisol axis is unlikely (cortisol at *T* = 0: <20 nmol/L; *T* = 30: 59 nmol/L; T = 60: 79 nmol/L).

In August 2017, an 8‐hour trough level prednisolone measurement of 18 µg/L (target range: 15‐25 µg/L) confirmed that his appropriate replacement dose is 4 mg once daily.

The patient remains well on 4 mg prednisolone daily and will continue on this indefinitely. This patient's experience confirms recent evidence that the dose required for adequate replacement is far less than the 7.5 mg traditionally quoted. If prednisolone is to be used as replacement glucocorticoid, we recommend a starting dose of 3‐4 mg daily.^1^

## 3 DISCUSSION

It is well known that patients with adrenal insufficiency should be given adequate steroid cover, especially at times of intercurrent illness. This is ingrained into doctors across all specialities given the lethal consequences of under‐replacement. Patients who do not receive adequate treatment are at risk of adrenal crises, presenting with vomiting, diarrhea, abdominal pain, lethargy, hypotension, hypoglycemia, and electrolyte dysregulation. Because of this, most patients are over‐replaced, and there are little data on the minimum dose of steroid required. Patients with pituitary failure have an intact adrenal gland and have normal regulation of aldosterone, and thus are not at risk of a salt‐losing crisis. Instead, when cortisol‐deficient, they develop exhaustion and eventually vomiting, but no life‐threatening salt‐losing crises.

Our evidence base for the dose of hydrocortisone replacement is poor. The average dose used as replacement has fallen from an average of 30 mg hydrocortisone daily to 20 mg daily, and the actual required dose is probably even lower than this. Although short‐term extra hydrocortisone may be lifesaving, chronic minor excess of replacement contributes to side effects such as osteoporosis.

The Endocrine Society Clinical Practice Guidelines endorse prednisolone as an alternative steroid for glucocorticoid replacement therapy.^2^ Taken only once a day, it is more convenient than hydrocortisone therapy and better mimics the normal cortisol day profile.

The association of high‐dose prednisolone with adverse metabolic outcomes such as osteoporosis has hindered wider adoption as a primary replacement therapy. This association is however based on studies in which prednisolone was used at doses in the order of 5‐7.5 mg daily for replacement, and much larger doses for immunosuppression.^3^ There is now evidence that prednisolone is more potent than previously thought and that a lower dose in the order of 2‐4 mg is in fact more appropriate.^4^, ^5^

## 4 CONCLUSION

Since this patient illustrated the safety of once‐daily prednisolone, we have changed to using prednisolone once daily as our first‐line glucocorticoid replacement therapy in patients with Addison's disease as well as (secondary) pituitary hypoadrenalism. We now have over 100 patients, who are currently treated with low‐dose (2‐4 mg) prednisolone as steroid replacement therapy.^1^ Those with adrenal failure also start on fludrocortisone 100 µg daily. Having developed a sensitive and reliable in‐house prednisolone assay, we have observed that there is variability in prednisolone metabolism between patients. We are able to tailor doses to each individual, using an 8‐hour trough measurement aiming for a level of 15‐25 µg/L. The majority of daily maintenance doses are between 2 and 4 mg once daily, with no observed adrenal crises thus far.

Clinicians hold a clear association between hypoadrenalism and hydrocortisone, meaning that steroid replacement therapy is less likely to be overlooked than with prednisolone. As the number of patients taking prednisolone for hypoadrenalism increases, it is important that this same association is entrained to prevent replacement therapy being inadvertently withheld.

## CONFLICT OF INTEREST

None declared.

## AUTHOR CONTRIBUTION

SC and PM: was responsible for the initial draft of this manuscript. KM and TT: have reviewed this manuscript and made edits to the text. SC, PM, TT and KM: have approved the final manuscript. All authors have contributed equally. KM: is the named physician who is responsible for this patient's care.

## References

[1] Smith D , Prabhudev H , Choudhury S , Meeran K . Prednisolone has the same cardiovascular risk profile as hydrocortisone in glucocorticoid replacement. Endocr Connect. 2017;6(8):766‐772. 29018153 10.1530/EC-17-0257 PMC5682416

[2] Bornstein SR , Allolio B , Arlt W , et al. Diagnosis and treatment of primary adrenal insufficiency: an endocrine society clinical practice guideline. J Clin Endocrinol Metab. 2016;101(2):364‐389. 26760044 10.1210/jc.2015-1710 PMC4880116

[3] Jodar E , Valdepenas MP , Martinez G , Jara A , Hawkins F . Long‐term follow‐up of bone mineral density in addison's disease. Clin Endocrinol (Oxf). 2003;58(5):617‐620. 12699444 10.1046/j.1365-2265.2003.01761.x

[4] Williams EL , Choudhury S , Tan T , Meeran K . Prednisolone replacement therapy mimics the circadian rhythm more closely than other glucocorticoids. J Appl Lab Med. 2016;1(2):152‐161. 10.1373/jalm.2016.020206 33626793

[5] Caldato MC , Fernandes VT , Kater CE . One‐year clinical evaluation of single morning dose prednisolone therapy for 21‐hydroxylase deficiency. Arq Bras Endocrinol Metabol. 2004;48(5):705‐712. 15761542 10.1590/s0004-27302004000500017

